# Genome Sequence of a Multidrug-Resistant Strain of *Stenotrophomonas maltophilia* with Carbapenem Resistance, Isolated from King Abdullah Medical City, Makkah, Saudi Arabia

**DOI:** 10.1128/genomeA.01166-15

**Published:** 2015-10-15

**Authors:** Alyaa M. Abdel-Haleem, Zineb Rchiad, Babar K. Khan, Abdallah M. Abdallah, Raeece Naeem, Shalam Nikhat Sheerin, Victor Solovyev, Abdalla Ahmed, Arnab Pain

**Affiliations:** a Biological and Environmental Sciences and Engineering (BESE) Division, King Abdullah University of Science and Technology (KAUST), Thuwal, Saudi Arabia; b Microbiology Unit, Department of Laboratory and Blood Bank, King Abdullah Medical City, Makkah, Saudi Arabia; c Department of Microbiology, College of Medicine, Umm Al-Qura University, Makkah, Saudi Arabia; d Computational Bioscience Research Centre, King Abdullah University of Science and Technology (KAUST), Thuwal, Saudi Arabia; e Softberry, Inc., Mount Kisco, New York, USA

## Abstract

The emergence and spread of multidrug-resistant (MDR) bacteria have been regarded as major challenges among health care-associated infections worldwide. Here, we report the draft genome sequence of an MDR *Stenotrophomonas maltophilia* strain isolated in 2014 from King Abdulla Medical City, Makkah, Saudi Arabia.

## GENOME ANNOUNCEMENT

*Stenotrophomonas maltophilia* is an emerging multidrug-resistant opportunistic pathogen with global prevalence (1). The increasing incidence of nosocomial and community-acquired *S. maltophilia* infections is of particular concern for immunocompromised individuals, as this bacterial pathogen is associated with a significant fatality/case ratio (1). The reported mortality rate for *S. maltophilia* ranges from 21 to 69% (2–4). Infections are intensified in environments of extreme temporal and spatial crowding. Therefore, during the pilgrimage season, Makkah, Saudi Arabia, is at high risk for rise of infections (5–7).

In this study, a multidrug-resistant (MDR) *S. maltophilia* strain was isolated from a patient at a tertiary-care hospital in Makkah, Saudi Arabia. The strain exhibited resistance to β-lactams, cephalosporins, carbapenems, aminoglycosides, fluoroquinolones, tetracyclines, polymyxin, and trimethoprim. Intermediate resistance to gentamicin and tigecycline was exhibited.

The genome of the isolated MDR *S. maltophilia* strain was sequenced on an Illumina HiSeq 2000 platform, using 3,106,105 paired-end 101-bp reads. The cleaned reads were assembled using the OligoZip pipeline (Softberry, Mount Kisco, NY) into 34 contigs (>1,000 bp), with a contig *N*_50_ of 198,115 bp. The draft genome assembly of MDR *S. maltophilia* is composed of 4,386,843 bp, with a G+C content of approximately 66.51%, and 4,047 protein-coding genes. Genome annotation was performed using the in-house annotation pipeline fgenesb.

Scanning of the Comprehensive Antibiotic Resistance Database (CARD) (8) with the assembled genome highlighted 30 hits, which include *smeE, smeB, acrB, acrD, mexB, acrF, smeC, smeF, smeS, smeD, smeA, smeR,* and *emrR.*

### Nucleotide sequence accession numbers.

The draft genome sequence of the MDR *S. maltophilia* strain has been deposited in the European Nucleotide Archive (ENA) under the accession numbers CWHS01000001 to CWHS01000034.
